# Effects of dietary nitrogen and/or phosphorus reduction on mineral homeostasis and regulatory mechanisms in young goats

**DOI:** 10.3389/fvets.2024.1375329

**Published:** 2024-05-10

**Authors:** Luisa S. Zillinger, Karin Hustedt, Nadine Schnepel, Frank Hirche, Marion Schmicke, Gabriele I. Stangl, Alexandra S. Muscher-Banse

**Affiliations:** ^1^Institute for Physiology and Cell Biology, University of Veterinary Medicine Hannover, Foundation, Hannover, Germany; ^2^Institute of Agricultural and Nutritional Science, Martin Luther University Halle-Wittenberg, Halle, Germany; ^3^Clinic for Cattle, University of Veterinary Medicine Hannover, Foundation, Hannover, Germany

**Keywords:** CYP27B1, dietary nitrogen and phosphate, FGFR1c, goat, vitamin D metabolism

## Abstract

**Introduction:**

The reduction of nitrogen (N) and phosphorus (P) in ruminant feed is desirable due to costs and negative environmental impact. Ruminants are able to utilize N and P through endogenous recycling, particularly in times of scarcity. When N and/or P were reduced, changes in mineral homeostasis associated with modulation of renal calcitriol metabolism occurred. The aim of this study was to investigate the potential effects of dietary N- and/or P-reduction on the regulatory mechanisms of mineral transport in the kidney and its hormonal regulation in young goats.

**Results:**

During N-reduction, calcium (Ca) and magnesium (Mg) concentrations in blood decreased, accompanied by a lower protein expression of cytochrome P450 family 27 subfamily B member 1 (CYP27B1) (*p* = 0.016). The P-reduced fed goats had low blood phosphate concentrations with simultaneously high Ca and Mg levels. The insulin-like growth factor 1 concentrations decreased significantly with P-reduction. Furthermore, gene expression of *CYP27B1* (*p* < 0.001) and both gene (*p* = 0.025) and protein (*p* = 0.016) expression of the fibroblast growth factor receptor 1c isoform in the kidney were also significantly reduced during a P-reduced diet. ERK1/2 activation exhibited a trend toward reduction in P-reduced animals. Interestingly, calcitriol concentrations remained unaffected by either restriction individually, but interacted significantly with N and P (*p* = 0.014). Additionally, fibroblast growth factor 23 mRNA expression in bone decreased significantly with P-restriction (*p* < 0.001).

**Discussion:**

These results shed light on the complex metabolic and regulatory responses of mineral transport of young goats to dietary N and P restriction.

## 1 Introduction

The reduction of nitrogen (N) and phosphorus (P) in feed for livestock is a much-discussed topic in environmental and resource protection. Excessive N and P content in animal feed not only causes high production costs, but also poses a significant environmental problem, e.g., through nutrient runoff, which leads to eutrophication of water bodies. Due to the ruminohepatic urea cycle, ruminant microorganisms can utilize N for microbial protein synthesis compared to monogastric species (1). Furthermore, ruminants can maintain P homeostasis even with reduced P-intake. This is due to their ability to recycle endogenous phosphate (P_i_) (2, 3). Nevertheless, an adequate supply of N and P is necessary for ruminants to maintain body functions.

Previous studies have shown that goats fed reduced N diets had lower blood calcium (Ca) levels (4, 5). In a study by Muscher and Huber (4), N-reduction also resulted in alterations in vitamin D metabolism. P-reduction also resulted in modulation of mineral homeostasis in goats and sheep with reduced plasma concentration of P_i_ and concomitant high plasma Ca levels in the blood (6, 7). The homeostasis of P_i_ and Ca in the body is tightly regulated by various hormones, primarily controlled by the interaction of active vitamin D (1,25(OH)_2_D_3_, calcitriol), parathyroid hormone (PTH), and fibroblast growth factor 23 (FGF23) (8). The enzyme cytochrome P450 family 27 subfamily B member 1 (CYP27B1) catalyzes the hydroxylation of calcidiol (25(OH)D_3_) to calcitriol in the kidney (9). Reduced plasma P_i_ concentration in monogastric species (10) and dairy cows (2) stimulates the synthesis of calcitriol. This results in an increased absorption of P_i_ from the intestine, where P_i_ transport proteins are upregulated (11). Conversely, elevated Ca concentrations inhibit the synthesis of calcitriol, leading to reduced Ca absorption from the intestine and the release of Ca and P_i_ from bone in both monogastric species (11, 12) and goats (13).

The degradation of calcitriol is facilitated by the enzyme cytochrome P450 family 24 subfamily A member 1 (CYP24A1). Additionally, CYP24A1 can convert calcidiol into 24,25-dihydroxyvitamin D_3_ (24,25(OH)_2_D_3_), another inactive form of vitamin D (14). Thus, calcitriol plays an essential role in maintaining serum Ca and P_i_ levels. Consequently, one of its effects is the stimulation of FGF23 secretion from osteoblasts and osteocytes (8). FGF23 primarily regulates plasma P_i_ concentration (8, 15), as demonstrated in sheep fed a P-reduced diet, where a simultaneous lower FGF23 mRNA expression in bone was observed (16). Therefore, FGF23 governs renal P_i_ excretion or reabsorption through the sodium-dependent P_i_ -transporters NaPiIIa (SLC34A1), NaPiIIc (SLC34A3) and possibly PiT2 (SLC20A2). However, no evidence of FGF23-dependent regulation was found for PiT1 (SLC20A1), another important sodium-dependent P_i_ transporter in the kidney (17). In contrast, in mature ruminants, renal P_i_ excretion does not appear to be influenced by low dietary P intake (18). FGF23 also modulates the expression of CYP27B1 in the kidneys of rodents (19). To exert these modulatory effects in the kidney, FGF23 requires its kidney-specific fibroblast growth factor receptor 1c (FGFR1c) and additionally its co-receptor α-Klotho for high-affinity binding to its receptor (20). Binding of FGF23 to this complex and subsequent activation of the FGFR1c/α-Klotho pathway triggers phosphorylation of the intracellular mitogen-activated protein kinase 3 and 1 (ERK1 and ERK2) signaling pathway (21). This activation then leads to suppression of renal expression of NaPiIIa, NaPiIIc and potentially PiT2 (17).

The aim of this study is to investigate the influence of an N- and/or P-reduced diet on the regulatory mechanisms of mineral transport in the kidneys of young goats, considering anticipated ruminant-specific alterations in these transport mechanisms. Previous research conducted by our team revealed lower concentrations of calcitriol and mRNA expression of CYP27B1 in goats fed an N-reduced diet (4, 22). Therefore, it was hypothesized that diminished levels of insulin-like growth factor 1 (IGF1) in plasma were responsible for the reduced expression of CYP27B1 and consequently calcitriol (22). However, the reduced IGF1 levels could also stimulate the release of FGF23 from bone, thereby inhibiting CYP27B1 expression. Conversely, reducing P intake might inhibit the release of FGF23 from bone, potentially leading to increased renal synthesis of calcitriol by stimulation CYP27B1 expression in young goats. Consequently, it will be investigated whether the observed lower CYP27B1 mRNA expression during N-reduction is corroborated at the protein level and maintained during simultaneous N- and P-reduction. Additionally, we aim to determine whether the hypothesized stimulatory effect of P-reduction on CYP27B1 expression outweighs the effects of N reduction.

## 2 Materials and methods

### 2.1 Ethical standards

The animal feeding and handling protocol for this experiment were approved by the Animal Welfare Officer of the University of Veterinary Medicine Hannover, Foundation (Hannover, Germany) and complied with the German Animal Welfare Law (permit number: 33.19–42,502–04-19/3076; Lower Saxony State Office for Consumer Protection and Food Safety (LAVES); 15 March 2019).

### 2.2 Animals and feeding regimes

For this molecular study, organs and blood samples were obtained from animals of a previous study (23) to avoid unnecessary repetition of animal model experiments. In brief, 28 male colored German goats from a commercial goat farm were selected for this experiment. This breed ensured uniform characteristics in terms of age, place of birth and genetic background, thus ensuring the reliability and reproducibility of the results. In addition, the same breed of goat was used in previous studies at this institute, which enables a direct comparison and continuity of the research results. The goats were initially fed a commercial milk replacer for 4 weeks, which was then slowly replaced by the control diet. After this adaptation period, the animals were divided into four different feeding groups and received (1) a control diet (16.48% crude protein (CP), 0.48% P, 1.3% Ca), (2) an N-reduced diet (8.35% CP, 0.51% P, 1.2% Ca), (3) a P-reduced diet (16.86% CP, 0.11% P, 1.2% Ca) or (4) an N- and P-reduced diet (8.1% CP, 0.11% P, 1.2% Ca). The level of urea was chosen to be underneath the threshold concentration at which the upregulation of rumen transepithelial urea transport begins. According to a previous study from our institute (24), the protein content was reduced to achieve a urea content of approximately 1 mmol/L in the blood. The amount of P in the diet was reduced to such extend that the P_i_ concentration in blood was approximately 1 mmol/L (25). The maximum amount of Ca in the diet for goats is suggested to be 1.5% of diet dry matter (DM) (26). In accordance with this recommendation, the Ca content in this study was adjusted to a maximum of 1.43% of DM. Individual feeding took place daily at 08.00 and 16.00 for 6 to 8 weeks. At the start of the experiment, the animals were 10 weeks old with an initial body weight (BW) of 19.04 kg (SEM 0.08) (23). A feed manufacturer specializing in feed production for animal research (ssniff Spezialdiäten GmbH, Soest, Germany) manufactured the pellets for the diets. All diets were isoenergetic, and contained approximately 12.7 MJ ME/kg DM. For further details on ingredients and chemical composition of the diets, see Weber et al. (23). Each feeding group consisted of seven animals housed together and received water *ad libitum*. Each animal received the amount of 55 g/kg^0.75^ pelleted concentrate twice a day. In addition, 25% of the concentrate weight was fed as wheat straw. To calculate the average intake of nutrients and minerals per animal, all feed offered and refused were recorded daily. The BW of the animals was measured weekly.

### 2.3 Body fluid and tissue sampling

Shortly before euthanasia, the goats underwent exsanguination following a standard abattoir captive bolt stunning procedure (Annex IV Directive 2010/63/EU). Blood samples were collected from the Vena jugularis using lithium-heparin syringes and serum syringes (Sarstedt AG & Co. KG, Nümbrecht, Germany). To mitigate diurnal variations, samples were consistently taken from fasting animals at the same morning time. Plasma and serum samples were centrifuged (2,000 g at room temperature, 15 min) for separation and subsequently stored at −20°C until further analysis. For technical reasons (Ussing chamber experiments), two goats per day were euthanized. To minimize potential time-related effects, animals from two feeding groups were slaughtered alternately. Within 5 min postmortem, the kidneys were removed, and tissue samples were taken from the cortex. The rib bones were dissected, the flesh was removed and then cut into cortical and medullary sections. The kidney cortices and bone sections were then rinsed with ice-cold saline (0.9% NaCl, w/v), immediately frozen in liquid N_2_ and stored at −80°C until further analysis.

### 2.4 Biochemical determinations

To determine the N, P_i_ and Ca status of the animals, the blood plasma concentrations of urea (commercial kit, R-Biopharm AG, Darmstadt, Germany), P_i_, and Ca were measured colorimetrically using standard spectrometric methods (27, 28) [interassay CV 3.79% (N), 1.88% (Ca) and 5.05% (P_i_); intraassay CV 1.2% (N), 2.85% (Ca) and 1.42% (P_i_)]. For an indirect assessment of PTH levels, plasma cyclic adenosine monophosphate (cAMP) was determined using competitive ELISA (Enzo Life Sciences GmbH, Lörrach, Germany). Serum concentrations of calcidiol and calcitriol were determined commercially either by HPLC or competitive ELISA (Immundiagnostik AG, Bensheim, Germany). Plasma concentration of 24,25(OH)_2_D_3_ was measured using liquid chromatography–tandem mass spectrometry at the Institute for Agricultural and Food Sciences at the Martin-Luther-University Halle-Wittenberg, Halle, Germany as described elsewhere (29). To analyze plasma growth hormone (GH), serum IGF1, plasma triiodothyronine (T3) and serum magnesium (Mg) concentrations, assays were conducted at the Clinic for Cattle, Laboratory of Endocrinology, University of Veterinary Medicine Hannover, Foundation. Techniques included in-house enzyme-linked immunosorbent assay, radioimmunoassays (Beckman Coulter GmbH, Krefeld, Germany) (30) or a standardized spectrometric technique. The concentration of blood glucose was measured in whole blood samples using the mutant Q-GDH-based blood glucose monitor method with an Accu-Chek Performa blood glucose meter (Roche Diagnostics GmbH, Mannheim, Germany).

### 2.5 Quantitative real-time PCR

Renal cortical tissue RNA was isolated using the RNeasy Plus Mini-Kit (Qiagen GmbH, Hilden, Germany), while bone tissue RNA was isolated using the RNeasy Fibrous Tissue Mini-Kit (Qiagen GmbH). Both kits contained spin columns to eliminate genomic DNA and were used in accordance with the manufacturer’s protocol. The RNA concentrations were measured spectrophotometrically using NanoDrop One (Thermo Fisher Scientific Inc., Waltham, MA, USA). Additionally, the quality and integrity of the RNA were assessed using an RNA 6,000 nanoassay on an Agilent 2,100 Bioanalyzer (Agilent Technologies Deutschland GmbH, Böblingen, Germany). Reverse transcription of 200 ng isolated RNA was conducted using random hexamers, oligo-dT primers, and TaqMan™ Reverse Transcription Reagent (Thermo Fisher Scientific Inc.) in accordance with the manufacturer’s protocol.

To evaluate mRNA expression of *NaPiIIa* in renal cortical tissue, caprine gene specific TaqMan primers and probes were synthesized by TIB Molbiol Syntheselabor GmbH (Berlin, Germany; Table 1). Each reaction mixture of 20 μL contained TaqMan™ Gene Expression Master Mix (Thermo Fisher Scientific Inc.), 16 ng reverse transcript complementary DNA (cDNA), 300 nmol/L specific primers, and 100 nmol/L specific probe. PCR products were amplified (50°C, 2 min; 95°C, 10 min; 40 cycles of 95°C, 15 s and 60°C, 1 min) and analyzed on a real-time PCR cycler (CFX96™, Bio-Rad Laboratories GmbH, Feldkirchen, Germany). Expression levels of *CYP24A1, CYP27B1, ERK1, ERK2, FGFR1c, α-Klotho*, sodium-potassium adenosine triphosphatase (*Na^+^/K^+^-ATPase*), *NaPiIIc, PiT1, PiT2* and xenotropic polytropic retrovirus receptor 1 (*XPR1*) in renal cortical tissue were quantified using SYBR Green® PCR assays with specific primers (Table 2). The expression of FGF23 in bone tissue was also quantified using SYBR Green® PCR assays with specific primers (Table 2). Reaction mixtures of 20 μL contained SensiFAST™ SYBR No-Rox Mix (BioCat GmbH, Heidelberg, Germany), 200 nmol/L specific primers, and 16 ng reverse transcribed cDNA. A real-time PCR cycler (Bio-Rad Laboratories GmbH) was used for amplification (3 min at 95°C; 40 cycles of 10 s at 95°C and 30 s at 60°C) and detection of PCR products. To determine the melting curve, the thermal profile began with an incubation of 10 min at 55°C with a gradual increase in temperature (0.5°C per 10 s) up to 95°C. Absolute copy numbers were determined using calibration curves generated with cloned PCR fragment standards (35). Specificity of amplicons was verified by sequencing (Microsynth Seqlab GmbH, Göttingen, Germany) and using NCBI Blast^1^. For renal cortical tissue, the reference gene 18S rRNA was synthesized by TIB MOLBIOL (Table 2). Additionally, reference genes beta-2-microglobulin (*B2M*), ribosomal protein L19 (*RPL19*), and ribosomal protein S9 (*RPS9*) were quantified with SYBR Green® PCR assays (Table 2). Among these, *RPL19* was identified as the most suitable reference gene for normalization. In bone tissue, the reference genes *18S*, *RPL19* and *RPS9* were synthesized as described. In bone tissue, *18S* rRNA was identified as the optimal reference gene for normalization. Gene expression was quantified with SYBR Green® PCR assay as described above. NormFinder software^2^ was used to determine the best reference gene for normalization. Each reaction was run twice and included water as a no-template control to ensure accuracy and reliability.

**Table 1:** Primers and probes used for TaqManTM assays in kidney cortex and bone tissue from young goats.

Gene^1^	Primers and probes (5′ ➔ 3′)	Accession number	References
NaPiIIa	Forward: CCACCGTACACGACTGCTTTAA Reverse: CATTTCTCCAGGGTGGCATT FAM-TGGCTGTCAGTTCTGGTGCTGCT-BBQ	XM_018050614.1 XM_018050615.1	(18)
18S rRNA	Forward: AAAAATAACAATACAGGACTCTTTCG Reverse: GCTATTGGAGCTGGAATTACCG FAM-TGGAATGAGTCCACTTTAAATCCTTCCGC-BBQ	AM711869.1	(31)

^1^NaPiIIa, sodium phosphate cotransporter type IIa; 18S rRNA, 18S ribosomal RNA.

**Table 2:** Primers used for SYBR green assays in kidney cortex and bone tissue from young goats.

Gene^1^	Primers and probes (5′ ➔ 3′)	Accession number	References
B2M	Forward: CCTTGGTCCTTCTCGGGCTG Reverse: TCTGGCGGGTGTCTTGAGTAT	XM_018053818.1	(32)
CYP24A1	Forward: GAGGCCTCAAGAAACAGCAC Reverse: CTGACCCTCTGCCAGTCTTC	XM_013968985.2 XM_018058054.1	(18)
CYP27B1	Forward: ACCTGGAAATTCCCGTGTCC Reverse: GATGCTTCTCTCAGGCACCA	XM_005680264.3	(18)
ERK1	Forward: CCCAAAGCTCTTGACCTGCT Reverse: AAGGTGAAAGGTTCCTCGGC	XM_018040780.1	This study
ERK2	Forward: TCCCGAATGCTGACTCCAAA Reverse: CTTGGGCAAGTCATCCAAATACA	NM_001314202.1	(33)
FGF23	Forward: TCCATGGAGACGGGCACATA Reverse: TGTGAAGTCCATGCAGAGGT	XM_018049064.1	(16)
FGFR1c	Forward: GCCAAACGTTGGACCAAGAC Reverse: AGCAGGCAAAACCAATGCAG	XM_018041769.1 to XM_018041782.1	(31)
α-Klotho	Forward: CACACAGCCCAGGACAATCT Reverse: CTCAAACTGATTTGCCGCGT	XM_018056638.1	(31)
Na^+^/K^+^-ATPase	Forward: TGGAACTCGGAGAAGAAGGA Reverse: GCCACTCGGTCCTGATATGT	XM_005690616.3	(22)
NaPiIIc	Forward: CTGCCTCGTCCTCATTGTCA Reverse: AGTGTTGGAACCCAGGAAGAG	XM_018056238.1 to XM_018056242.1	This study
PiT1	Forward: ATTCATCCTCCGTAAGGCGGATC Reverse: CAGCAATGGTGCTCCAGTGTACA	XM_018055327.1	(22)
PiT2	Forward: CCAATCTCGGGGACTCACTG Reverse: GGAACGGGGTCCTCCTTTTT	XM_018041866.1 to XM_018041872.1	(6)
RPL19	Forward: AGCCTGTGACTGTCCATTCC Reverse: ACGTTACCTTCTCAGGCATT	XM_005693740.3	(32)
RPS9	Forward: CGCCTCGACCAAGAGCTGAAG Reverse: CTCCAGACCTCACGTTTGTTCC	XM_018063497.1	(34)
XPR1	Forward: AATGCCGATGATCAGACGCT Reverse: AGCCTTGGATTGAGAAGCGA	XM_018060672.1	(6)

^1^B2M, beta-2-microglobulin; CYP24A1, cytochrome P450 family 24 subfamily A member 1; CYP27B1, cytochrome P450 family 27 subfamily B member 1; ERK1, mitogen-activated protein kinase 3; ERK2, mitogen-activated protein kinase 1; FGF23, fibroblast growth factor 23; FGFR1c, fibroblast growth factor receptor 1c isoform; Na^+^/K^+^-ATPase, sodium-potassium adenosine triphosphatase; NaPiIIc, sodium phosphate cotransporter type IIc; PiT1, sodium-dependent P_i_ transporter 1; PiT2, sodium-dependent P_i_ transporter 2; RPL19, ribosomal protein L19; RPS9, ribosomal protein S9; XPR1, xenotropic and polytropic retrovirus receptor 1.

### 2.6 Tissue extraction and western blot analysis

To quantify the expression of NaPiIIa and PiT2, brush border membranes (BBM) were prepared in accordance with the method of Wilkens et al. (35). Cytosolic preparation was required for quantification of ERK1/2, whereas crude membranes (CM) were isolated for quantification of Na^+^/K^+^-ATPase, both in accordance with the method of Wilkens et al. (36). Total cell lysate preparation was required to determine CYP27B1 and FGFR1 protein expression in accordance with the method of Weber et al. (23). Protein concentrations of all cell fractions were measured using a commercial Bradford assay (Serva Elektrophoresis GmbH, Heidelberg, Germany). For detection of NaPiIIa protein expression, 15 μg of BBM was separated by 8.5% SDS-PAGE and transferred to nitrocellulose membranes (GE Healthcare Europe GmbH, Freiburg, Germany). The protein was incubated overnight with anti-NaPiIIa polyclonal antibody (Alpha Diagnostic Intl. Inc., San Antonio, TX, USA), diluted 1: 300 in phosphate-buffered saline (PBS) with 0.1% Tween 20 (PBST) and 2.5% non-fat milk powder at 4°C overnight. Immunoreactivity of the primary antibody was detected with an HRP-coupled secondary anti-rabbit antibody (diluted 1: 20,000). The specificity of the NaPiIIa antibody was successfully demonstrated by preincubation of the primary antibody with the corresponding antigenic peptide (data not shown). For detection of PiT2 protein expression, 25 μg of BBM was separated by 8.5% SDS-PAGE and transferred to nitrocellulose membranes (GE Healthcare Europe GmbH). Overnight incubation with a polyclonal anti-PiT2 antibody (self-made, Davids Biotechnologie GmbH, Regensburg, Germany, gift from Prof. V. Sorribas, Laboratory of Molecular Toxicology, University of Zaragoza, Spain) was performed, diluted 1: 1,000 in PBST. An HRP-coupled secondary anti-rabbit antibody (diluted 1: 2,000) was used to detect immunoreactivity of the primary antibody. To quantify ERK1/2 protein expression, 10 μg of cytosol was separated by 8.5% SDS-PAGE and transferred to nitrocellulose membranes (GE Healthcare Europe GmbH). To determine the level of phosphorylation, a primary anti-ERK1/2 (Cell Signaling Technology Europe B. V., Frankfurt am Main, Germany) was first incubated overnight and diluted 1: 2,000 in 5% bovine serum albumin (BSA) buffer and tris-buffered saline (TBS) with 0.1% Tween 20 (TBST). Immunoreactivity of the phosphospecific antibody was detected using an anti-rabbit antibody (diluted 1:2,500). The antibody was then removed by incubating the membrane with stripping buffer (pH 2.0). The membrane was blocked and incubated again overnight with the non-phosphospecific primary anti-ERK1/2 antibody (cell signaling), diluted 1:1,000 in 5% BSA and TBST. Immunoreactivity of the primary antibody was detected with anti-rabbit antibody (diluted 1: 2,500). The monoclonal ERK1/2 antibody recognizes bovine ERK1/2. For Na^+^/K^+^-ATPase, 10 μg of CM were separated by 8.5% SDS-PAGE and transferred onto nitrocellulose membranes (GE Healthcare Europe GmbH). To detect the Na^+^/K^+^-ATPase protein, the membranes were incubated overnight at 4°C with an anti- Na^+^/K^+^-ATPase antibody (Enzo Life Sciences GmbH) diluted 1: 10,000 in TBST and 5% non-fat milk powder. Immunoreactivity of the primary antibody was detected with an HRP-coupled anti-mouse antibody (diluted 1: 20,000). The monoclonal Na^+^/K^+^-ATPase antibody recognizes sheep and bovine Na^+^/K^+^ ATPase. For the quantification of CYP27B1, 10 μg of cell lysate was used. Membranes were incubated overnight with caprine anti-CYP27B1 (self-made, Davids Biotechnologie GmbH), diluted 1:500 in TBST and 2.5% non-fat milk powder. An HRP-coupled anti-rabbit antibody (diluted 1: 20,000) was used to detect the primary antibody. The specificity of the goat CYP27B1 antibody was successfully demonstrated by preincubation of the primary antibody with the corresponding antigenic peptide (data not shown). For the determination of FGFR1 protein, 25 μg of cell lysate was separated by 8.5% SDS-PAGE and transferred onto nitrocellulose membranes (GE Healthcare Europe GmbH). The protein was incubated overnight with a polyclonal anti-FGFR1 antibody (Thermo Fisher Scientific Inc.) 1:1,000 in TBST and 5% BSA. The primary antibody was detected with an HRP-coupled anti-rabbit antibody (diluted 1:2,500). The primary antibodies used were either specific to the particular caprine protein or exhibited species cross-reactivity for caprine, ovine or bovine target tissue. The protein sequences were verified for their homology with NCBI Blast (see text footnote 1). Immunoblot assays detecting the expression of ERK1/2, NaPi IIa and Na^+^/K^+^ ATPase proteins in caprine tissue were performed as described elsewhere (5, 37). Membranes were incubated solely with the secondary antibody to ensure exclusive detection of specific signals. For the detection of the specific proteins, a chemiluminescence system and the ChemiDoc MP (Bio-Rad Laboratories GmbH) were used. Densitometric measurements of proteins were performed using Image Lab 5.2.1 software (Bio-Rad Laboratories GmbH). For semi-quantification, the values of the quantified specific proteins were normalized to the amount of total protein stained with Indian ink. This normalization process aids in providing relative measurements of protein expression levels.

### 2.7 Statistical analysis

The sample size (*n* 7/group) was determined based on metabolic data from a previous study (5) with a statistical power of 0.8 and α error of 0.05. All data are given as means with their standard errors (SEM) unless otherwise stated. For data analysis, GraphPad Prism version 9.3 (GraphPad Software, San Diego, CA, USA) was used. All data were tested for normal distribution and were analyzed by two-way ANOVA with Tukey’s multiple comparisons test. *p* < 0.05 was considered significantly different, and *p* < 0.1 was used to define trends. Potential linear relationships between mRNA and protein expression levels, as well as correlations between gene and protein expression with blood metabolites, were calculated, using a simple correlation analysis with Pearson’s correlation coefficient. Non-linear relationships were calculated using Spearman’s correlation coefficient. Furthermore, potential outliers were tested using the ROUT method (38).

## 3 Results

### 3.1 Feed intake and growth performance of young goats fed a nitrogen- and/or phosphorus-reduced diet

All animals were clinically healthy throughout the study. The clinical health of the animals was ensured through monitoring and care by a qualified veterinarian that examined the animals daily and closely monitored their behavior and physical condition. The data on feed intake, daily weight gain and BW of the goats have been previously published (23). The data on feed intake are listed in Table 3. Daily BW gain was lower by 79% in the P-restricted group and by 64% in the N-/P-restricted group, resulting in final BW reductions of 20 and 16%, respectively, compared to the control group. The DM intake diminished by about 14% in the P- and 13% in the N-/P-groups. The concentrate intake diminished by about 14% in the P- and the N-/P- groups. The BW of goats in the N-restricted group remained unaffected. N-intake was lower by about 13% with P-restriction, 50% with N-restriction, and 56% with combined N- and P-restriction. Ca-intake was lower by 17% with P-restriction and 20% with N-/P-restriction. P-intake dropped by about 78% in both the P-restricted and N-/P- restricted groups.

**Table 3:** Mean daily intake of dry matter (DM), concentrate, nitrogen (N), phosphorus (P) and calcium (Ca) of growing goats fed an N- and/or P-reduced diet.

Items	N+/P+	N-/P+	N+/P-	N-/P-	*p*-value		
					N-reduction	P-reduction	Interaction
DM intake (g/d)*	614	594	528	534	0.707	0.001	0.485
	± 10^a^	± 6^a,b^	± 25^b^	± 25^b^			
Concentrate intake (g/d)*	555	537	469	475	0.799	0.002	0.581
	± 12^a^	± 7^a,b^	± 29^b^	± 28^a,b^			
N intake (g/d)*	13.39	6.76	11.64	5.87	<0.0001	0.003	0.308
	± 0.27^a^	± 0.09^b^	± 0.68^c^	± 0.33^b^			
P intake (g/d)*	2.40	2.49	0.53	0.54	0.189	<0.0001	0.282
	± 0.05^a^	± 0.03^a^	± 0.03^b^	± 0.03^b^			
Ca intake (g/d)*	6.50	6.20	5.42	5.22	0.274	0.0001	0.827
	± 0.13^a^	± 0.08^a,b^	± 0.31^b,c^	± 0.29^c^			

(Mean values with their standard errors; *n* = 7 animals).

^a,b,c^Mean values within a row with unlike superscript letters were significantly different; Tukey’s multiple comparisons test (*p* < 0.05).

*Data already published in Weber et al. (23).

### 3.2 Blood metabolites of young goats fed a nitrogen- and/or phosphorus-reduced diet

Blood metabolites are shown in Table 4. Some of these blood metabolites have already been published in Weber et al. (23). In the N-restricted feeding groups, blood plasma concentrations of urea were lower by about 83%. The P-reduced feeding groups showed significantly reduced blood plasma concentrations of P_i_ by about 67%, with concomitant higher blood Ca concentrations by about 37%. The glucose concentration in whole blood and the T3 concentration in plasma were consistent across all feeding groups (23). Plasma concentrations of cAMP was lower with N-restriction by trend and remained unaffected by P-restriction. Serum levels of calcidiol and calcitriol were not affected by N-reduction or P-reduction alone, but there was a significant interaction between N and P on calcitriol concentration. Blood plasma concentrations of 24,25(OH)_2_D_3_ were lower with the P-reduced diet, whereas an N-reduction had no effect on them. Plasma concentrations of GH were not affected by diet, whereas serum IGF1 concentrations tended to be lower with N-reduction and were significantly lower with P-reduction. Serum concentrations of Mg were higher with P-reduction and diminished by N-reduction.

**Table 4:** Blood metabolites of young growing male goats fed an N- and/or P-reduced diet.

Item^1^	N+/P+	N-/P+	N+/P-	N-/P-	SEM	*p*-value		
						N-reduction	P-reduction	Interaction
*n*	7	7	7	7				
Calcium (mmol/L)*	3.16^a^	3.11^a^	4.34^b^	3.55^a^	0.24	0.002	<0.001	0.005
Calcidiol (nmol/L)	47.71	53.57	56.86	50.71	6.38	0.965	0.335	0.072
Calcitriol (pg/mL)	18.46	14.57	13.39	18.76	3.48	0.673	0.801	0.014
cAMP (pmol/mL)	21.51	20.59	36.63	17.63‖	11.24	0.090	0.291	0.121
IGF1 (ng/mL)	243.8^a^	193.8^a^	123.8^b^	116.3^b^	32.38	0.090	<0.001	0.202
GH (ng/mL)	5.84	6.22	4.90	5.31	1.43	0.580	0.209	0.987
Glucose (mg/dL)*	68.00	67.86	67.43	66.57	4.19	0.814	0.662	0.866
Magnesium (mmol/L)	0.95^a^	0.72^b^	1.09^a,c^	1.01^a^	0.09	0.003	<0.001	0.120
Phosphate (mmol/L)*	2.09^a^	2.00^a^	0.70^b^	1.06^b,c^	0.26	0.301	<0.001	0.099
T3 (nmol/L)	1.83	2.04	1.94	1.94	0.28	0.464	0.943	0.446
Urea (mmol/L)*	6.76^a^	1.16^b^	6.85^a^	2.22^b,c^	0.83	<0.001	0.180	0.250
24,25(OH)_2_D_3_ (ng/mL)	8.58	8.68	4.88	7.29	2.47	0.318	0.050	0.361

(Mean values with their pooled standard errors; *n* = 6–7 animals).

^1^IGF1, Insulin-like growth factor 1; T3, Triiodthyronine 3.

^a,b,c^Mean values within a row with unlike superscript letters were significantly different; Tukey’s multiple comparisons test (*p* < 0.05).

*Data already published in Weber et al. (23).

‖ *n* = 6; due to the outlier test performed.

### 3.3 Effects of a nitrogen- and/or phosphorus-reduced diet on mRNA and protein expression of selected target genes in the kidney

**Figure 1:**
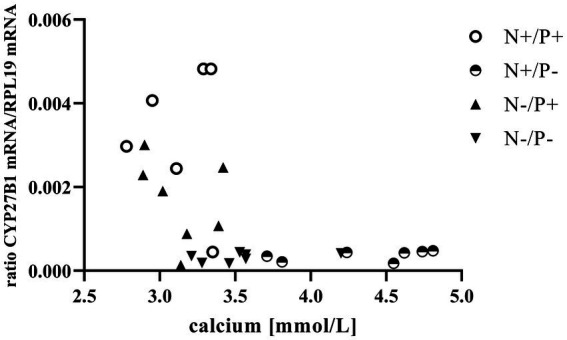
Relationship between blood calcium and cytochrome P450 family 27 subfamily B member 1 (*CYP27B1*) mRNA expression (*r* = −0.47, *p* = 0.014). The level of significance with Spearman’s correlation coefficient was set at *p* = 0.05.

**Figure 2:**
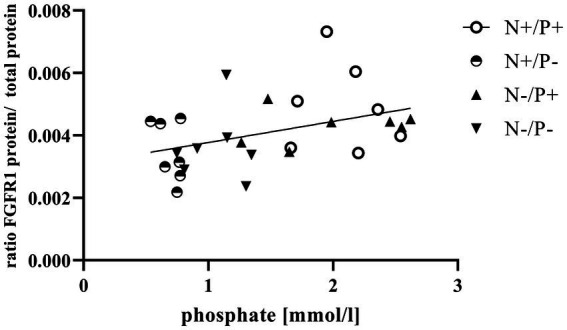
Linear relationship between phosphate in blood and fibroblast growth factor receptor 1 (FGFR1) protein expression (*r* = 0.42, *p* = 0.025). The level of significance with Pearson’s correlation coefficient was set at *p* = 0.05.

**Figure 3:**
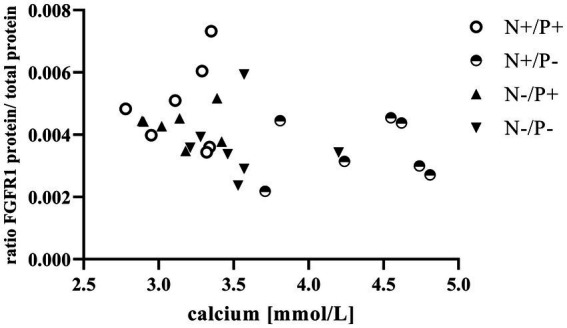
Relationship between calcium in blood and fibroblast growth factor receptor 1 (FGFR1) protein (*r* = −0.41, *p* = 0.031). The level of significance with Spearman’s correlation coefficient was set at *p* = 0.05.

**Figure 4:**
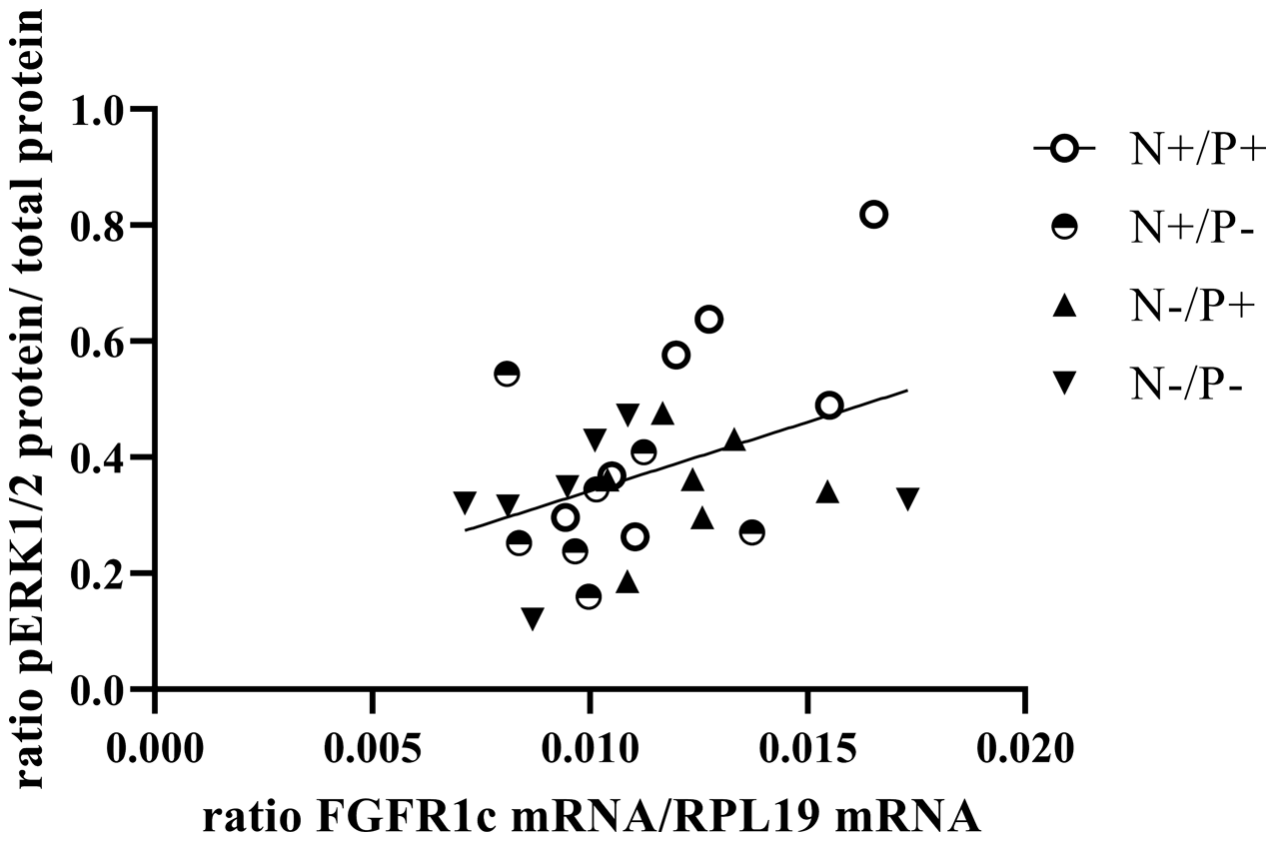
Linear relationship between phosphorylated mitogen-activated protein kinase 3/1 (pERK1/2) protein and fibroblast growth factor receptor 1c (*FGFR1c*) mRNA expression (*r* = 0.41, *p* = 0.029). The level of significance with Pearson’s correlation coefficient was set at *p* = 0.05.

The average RNA integrity number (RIN) of the kidney cortex tissue samples was 9.14 (SEM 0.12). The results of all genes and corresponding proteins expressed in the renal cortex are shown in Tables 5, 6. CYP27B1 mRNA expression was significantly reduced due to both N-reduction and P-reduction, as well as the interaction between N and P. Protein expression of CYP27B1 was lower in the N-restricted feeding group compared to the other feeding groups. Furthermore, CYP27B1 mRNA expression was negatively correlated with blood Ca concentration (*r* = −0.47, *p* = 0.014; Figure 1). FGFR1c mRNA and protein expression were significantly lower in the P-reduced feeding groups. FGFR1 protein expression correlated positively with plasma concentration of P_i_ (*r* = 0.42; *p* = 0.025; Figure 2) and negatively with plasma concentration of Ca (*r* = −0.41; *p* = 0.031; Figure 3). Furthermore, Na^+^/K^+^-ATPase mRNA expression was significantly lower in both groups receiving an N-reduced diet, while its protein expression remained unchanged. Na^+^/K^+^-ATPase mRNA expression was positively correlated with its protein expression. Although, NaPiIIa gene expression was not affected, NaPiIIa protein expression was higher in the P-reduced animals and tended to be higher in the N-reduced animals. The gene expression of NaPiIIc was lower by trend with P-restriction. Protein expression of ERK1/2 did not change, while pERK1/2 and the ratio of pERK1/2 to ERK1/2 showed a trend toward diminished expression in the P-reduced animals. Neither ERK1 nor ERK2 gene expression changed with different diets. Protein expression of ERK1/2 positively correlated with gene expression of FGFR1c (*r* = 0.41; *p* = 0.029; Figure 4). Expression of PiT2 mRNA showed a trend toward higher expression in the N-restricted feeding groups. However, protein expression of PiT2 was not affected by any of the feeding groups. The mRNA expression of CYP24A1, α-Klotho, PiT1 and XPR1 mRNA did not differ between the feeding groups.

**Table 5:** Relative levels of CYP24A1, CYP27B1, ERK1, ERK2, FGFR1c, α-Klotho, Na^+^/K^+^-ATPase, NaPiIIa, NaPiIIc, PiT1, PiT2 and XPR1-mRNA expression normalized to RPL19 in the renal cortex of young growing male goats fed an N- and/or P-reduced diet.

Item^1^	N+/P+	N-/P+	N+/P-	N-/P-	SEM	*p*-value		
						N-reduction	P-reduction	Interaction
*n*	7	7	7	7				
CYP24A1	16.75×10^−3^	12.08×10^−3^	20.51×10^−3^	18.31×10^−3^	0.009	0.448	0.273	0.784
CYP27B1	3.26×10^-3a^‖	1.68×10^-3b^	0.36×10^-3b^	0.32×10^-3b^	0.001	0.036	<0.001	0.045
ERK1	1.23×10^−3^	0.85×10^−3^	1.32×10^−3^	1.04×10^−3^	0.001	0.131	0.515	0.812
ERK2	0.38×10^−3^	0.36×10^−3^	0.37×10^−3^	0.33×10^−3^	0.001	0.393	0.503	0.816
FGFR1c	12.53×10^−3^	12.38×10^−3^	10.17×10^−3^	10.24×10^−3^	0.002	0.966	0.025	0.907
α-Klotho	0.50	0.49	0.56	0.51	0.132	0.705	0.551	0.792
Na^+^/K^+^-ATPase	2.16^a^	1.69^b^	1.97^a,b^	1.86^a,b^	0.213	0.011	0.935	0.099
NaPiIIa	30.77×10^−3^	25.51×10^−3^	25.84×10^−3^	29.34×10^−3^	0.008	0.836	0.897	0.309
NaPiIIc	56.18×10^−3^	57.45×10^−3^	51.55×10^−3^	48.04×10^−3^	0.008	0.785	0.099	0.564
PiT1	4.11×10^−3^	3.89×10^−3^	3.93×10^−3^	3.51×10^−3^	0.001	0.191	0.260	0.680
PiT2	1.83×10^−3^	1.85×10^−3^	2.19×10^−3^	2.11×10^−3^	0.001	0.063	0.838	0.772
XPR1	13.31×10^−3^	12.76×10^−3^	14.14×10^−3^	13.57×10^−3^	0.001	0.433	0.251	0.990

(Mean values with their pooled standard errors; *n* = 6–7 animals).

^1^CYP24A1, cytochrome P450 family 24 subfamily A member 1; CYP27B1, cytochrome P450 family 27 subfamily B member 1; ERK1, mitogen-activated protein kinase 3; ERK2, mitogen-activated protein kinase 1; FGFR1c, fibroblast growth factor receptor 1c isoform; Na^+^/K^+^-ATPase, sodium-potassium adenosine triphosphatase; NaPiIIa, sodium phosphate cotransporter type IIa; NaPiIIc, sodium phosphate cotransporter type IIc; PiT1, sodium-dependent P_i_ transporter 1; PiT2, sodium-dependent P_i_ transporter 2; XPR1, xenotropic and polytropic retrovirus receptor 1.

^a,b^Mean values within a row with unlike superscript letters were significantly different; Tukey’s multiple comparisons test (*p* < 0.05).

‖ *n* = 6; due to the outlier test performed.

**Table 6:** Relative amounts of CYP27B1, ERK1/2, pERK1/2, FGFR1, Na^+^/K^+^-ATPase, NaPiIIa and PiT2 protein expression in the kidney cortex of young growing male goats fed an N- and/or P-reduced diet.

Item^1^	N+/P+	N-/P+	N+/P-	N-/P-	SEM	*p*-value		
						N-reduction	P-reduction	Interaction
*n*	7	7	7	7				
CYP27B1	4.61×10^−3^	2.46×10^−3^	3.58×10^−3^	2.36×10^−3^	0.001	0.016	0.392	0.479
ERK1/2	13.26×10^-3a^	13.13×10^-3a,b^	12.75×10^-3a,c^	13.23×10^-3a^	0.001	0.584	0.542	0.365
pERK1/2	6.49×10^−3^	4.58×10^−3^	3.97×10^−3^	4.44×10^−3^	0.001	0.300	0.061	0.090
Ratio pERK1/2 to ERK1/2	0.49	0.35	0.32	0.33	0.105	0.246	0.079	0.146
FGFR1	4.90×10^−3^	4.30×10^−3^	3.49×10^−3^	3.65×10^−3^	0.001	0.584	0.016	0.348
Na^+^/K^+^-ATPase	12.98×10^-3a^	12.56×10^-3a^	13.44×10^-3a^	12.95×10^-3b^	0.001	0.495	0.525	0.956
NaPiIIa	0.81×10^-3a^	0.83×10^-3a,b^	0.86×10^-3a,c^	1.30×10^-3a^	0.001	0.056	0.021	0.036
PiT2	0.23×10^−3^	0.25×10^−3^	0.14×10^−3^	0.22×10^−3^	0.001	0.230	0.212	0.560

(Mean values with their pooled standard errors; *n* = 7 animals).

^1^CYP27B1, cytochrome P450 family 27 subfamily B member 1; ERK1/2, mitogen-activated protein kinase 3/1; FGFR1, fibroblast growth factor receptor 1; Na^+^/K^+^-ATPase, sodium-potassium adenosine triphosphatase; NaPiIIa, sodium phosphate cotransporter type IIa; PiT2, sodium-dependent P_i_ transporter 2.

^a,b,c,^Mean values within a row with unlike superscript letters were significantly different; Tukey’s multiple comparisons test (*p* < 0.05).

### 3.4 Effects of a nitrogen- and/or phosphorus-reduced diet on mRNA expression of FGF23 in bone

The average RIN of the bone tissue samples was 8.69 (SEM 0.12). The mRNA expression of FGF23 is presented in Table 7. The FGF23 mRNA expression was significantly lower with P-restricted feeding.

**Table 7:** Relative levels of FGF23 mRNA normalized to 18S rRNA in compact bone tissue of young growing male goats fed an N- and/or P-reduced diet (Mean values with their pooled standard errors; *n* = 7 animals).

Item^1^	N+/P+	N-/P+	N+/P-	N-/P-	SEM	*p*-value		
						N-reduction	P-reduction	Interaction
*n*	7	7	7	7				
FGF23	75.14×10^-5a^	68.6×10^-5a^	3.70×10^-5b^	8.86×10^-5b^	0.001	0.946	<0.001	0.576

^1^FGF23, fibroblast growth factor 23.

^a,b^Mean values within a row with unlike superscript letters were significantly different; Tukey’s multiple comparisons test (*p* < 0.05).

## 4 Discussion

This study focused on the influence of N- and/or P-reduction on mineral homeostasis and its hormonal regulation in young goats. To our knowledge, this is the first study in young goats to investigate the effects of a simultaneous N- and P-reduced diet at the molecular level in the kidney.

Hormonal regulation of P_i_ and Ca homeostasis in blood is maintained by the described interplay of calcitriol, FGF23 and PTH (8). In monogastric species, P-reduction stimulated calcitriol synthesis (39) due to increased CYP27B1 expression mediated by pituitary hormones. However, previous studies in adult sheep and in female lactating goats have shown that blood calcitriol concentrations were not changed by P-reduction (7, 40). In this study conducted with young goats, the observed unchanged GH concentrations do not provide evidence that pituitary hormones stimulate renal CYP27B1 expression during P-reduction. This is further supported by the finding of reduced CYP27B1 expression and unchanged calcitriol concentrations in the current study.

The observed negative correlation between blood Ca concentration and *CYP27B1* expression in this study indicates a relationship between the two variables (Figure 1). Elevated levels of circulating Ca are known to suppress CYP27B1 primarily by inhibiting PTH release (41). This reduction in calcitriol synthesis serves as a regulatory mechanism to prevent the animals from absorbing excessive Ca (11, 12).

An interesting result of this study is the increase in blood Mg concentration in P-reduction. In the kidney, Mg is passively reabsorbed paracellularly along its electrochemical gradient and actively reabsorbed transcellularly via Mg-permeable channels. In the current study, the increased Mg concentrations, like the increased Ca concentrations in the case of P-reduction, are presumably due to stimulated bone resorption. The increase in serum Mg during P-reduction, like the increased blood Ca levels mentioned earlier, could lead to an inhibition of PTH release from the parathyroid glands by activating the Ca sensing receptor. This would be an explanation for the reduced *CYP27B1* mRNA expression (42). Moreover, the lower Mg concentrations in the serum of goats with N-reduction, and the simultaneously lower Ca concentrations in the blood, aligns with findings observed in humans, as well (43).

The current study is the first to quantify the protein expression of CYP27B1 in goats. Interestingly, contrary to the gene expression findings in these goats, P-reduction had no measurable effect on CYP27B1 protein expression. This discrepancy suggests the presence of additional regulatory mechanisms between transcript and protein levels. In the N-reduced animals of the current study, the low gene expression of *CYP27B1* aligns with previous findings (31), which were also confirmed at protein level in this study. In the study by Wilkens et al. (31), the low levels of *CYP27B1* mRNA were probably due to reduced levels of IGF1 in the blood of young goats during an N-reduced diet. IGF1 levels not only have a direct influence on CYP27B1 expression in the kidney but also affect bone metabolism. In humans, the synthesis of FGF23 in bone is inhibited by various factors, including IGF1 and insulin (44). Since an inhibitor of FGF23 synthesis is diminished during a reduced N diet and especially during a reduced P diet, a stimulation of FGF23 synthesis would be expected. To assess the status of FGF23, its mRNA expression in bone tissue was examined in the young goats of this study because no goat-specific blood FGF23 assays are currently available. Thus, this study demonstrates that the lower levels of *CYP27B1* mRNA expression were not caused by stimulation of FGF23 expression from bone but were directly caused by lower IGF levels in blood, as initially hypothesized. The influence of IGF1 on CYP27B1 expression was also reflected in the higher significance of reduced IGF1 levels under P-restriction compared with the N-reduced animals. This was accompanied by a greater reduction in *CYP27B1* mRNA under P-restriction.

Enhanced FGF23 signaling in response to elevated blood P_i_ levels is a well-known effect in monogastric species (45). FGF23 lowers renal NaPiIIa and NaPiIIc expression and leads to lower blood P_i_ concentration *in vivo*, predominantly via FGFR1c (46). The synthesis of FGFR1c in the kidney is directly upregulated by FGF23 through unknown mechanisms (47). In this study, the positive correlation of plasma P_i_ with FGFR1 protein (Figure 2) confirms that FGFR1 protein expression could be regulated by P_i_ in goats as in monogastric animals. This explains the lower expression of the *FGF23* gene and the FGFR1(c) gene and protein in the P-reduced animals, whereas the expression of FGFR1(c) and *FGF23* was not affected in the N-reduced animals. The prominent high blood Ca levels in the P-reduced goats of the current study might have also been a reason for the reduced *FGF23* expression in the bones and FGFR1(c) in the kidneys. In FGF23 and FGFR1 knockout mice, Ca loss was observed due to a downregulation of the transient receptor potential cation channel subfamily V member 5 (48, 49). In these mice, this was regulated by a lower FGF23-induced ERK1/2 signaling cascade. In the present study, the interplay of Ca and FGF23 signaling is shown in an inverse correlation of plasma Ca with FGFR1 protein (Figure 3). Although FGFR1(c) and *FGF23* expression changed, *α-Klotho* mRNA remained unaffected by the different diets. This could be explained by the unaffected blood level of calcitriol in the goats of this study. In a previous study on Ca-restricted goats, the increased expression of *Klotho* mRNA was hypothesized due to the presence of a vitamin D-responsive element in the *Klotho* gene (31), as is the case for the human and mouse *Klotho* gene (50).

In the current study, P-reduction resulted in lower *FGF23* levels and thus decreased FGFR1c expression. This reduction also tended to reduce phosphorylation and subsequent activation of the ERK1/2 signaling cascade, just as in monogastric species (21). The positive correlation of *FGFR1c* mRNA and ERK1/2 protein (Figure 4) of the current study underlines this. Furthermore, a direct P-sensing mechanism, as postulated in various organs, could also be responsible for the tendentially reduced protein expression of pERK1/2 upon P-reduction (11).

The first step of calcitriol degradation is mainly mediated by the renal enzyme CYP24A1 through VDR (51). In addition, CYP24A1 also catalyzes calcidiol to 24,25(OH)_2_D_3_, an inactive form of vitamin D (14). To determine the degradation status of vitamin D in the goats of this study, blood concentrations of 24,25(OH)_2_D_3_ were measured during different intakes of N and/or P. The diminished blood concentration of 24,25(OH)_2_D_3_ during P-reduction indicates reduced catabolic degradation of calcidiol. In FGF23-overexpressing mice, CYP24A1 transcript levels were increased (19). Furthermore, FGF23-induced activation of FGFR1c in the kidney of monogastric animals resulted in higher CYP24A1 activation and consequently higher 24,25(OH)_2_D_3_ synthesis (52). Therefore, the low FGFR1 expression during P-reduction in this study could be responsible for the reduced 24,25(OH)_2_D_3_ concentration_._ Even though the gene expression of *CYP24A1* was not significantly altered in these goats, the activity of the enzyme might still be changed. Since no goat-specific antibody against the CYP24A1 protein is available, altered protein expression and reduced activity cannot be ruled out.

A limitation of the study is the lack of urine parameters derived from 24-h urine samples, which could provide valuable insights into the functional implications of the observed molecular changes. Additionally, investigating the effects of N- and/or P-reduced feeding in goats at later stages in life, particularly in female animals, would be of interest. The rumen and its microbial population exhibit remarkable adaptability, and considering the additional influence of sex hormones on mineral homoeostasis is crucial for a comprehensive understanding of these processes in ruminants.

One potential application of the findings of the current study is the development of customized feeding protocols tailored to the specific nutritional needs of goats at different life stages and production goals. By incorporating knowledge of how dietary N and P levels affect mineral metabolism, producers can formulate more precise rations that optimize nutrient utilization and promote overall goat health and performance. In addition, the identification of key genes and proteins involved in mineral transport and hormone regulation offers opportunities for the development of targeted nutritional supplements or feed additives aimed at influencing these metabolic pathways. Such interventions could help to address specific nutritional problems or deficiencies in commercial goat herds and ultimately increase productivity and profitability. Furthermore, the knowledge gained from this research underlines the importance of constant innovation and adaptation in goat feeding to meet new challenges such as environmental sustainability and animal welfare. By integrating scientific advances into practical management strategies, goat farmers can improve the efficiency and sustainability of their operations while ensuring the health and welfare of their animals. Although the experiments conducted in this study are basic research, the results have the potential to significantly advance goat feeding practices in the industry.

## 5 Conclusion

This study demonstrated that the lower CYP27B1 mRNA observed during N-reduced feeding in young goats was also evident at the protein level. The reduction in CYP27B1 expression was primarily attributed to lower levels of IGF1 during this dietary treatment and not by FGF23 from bone. Although reduced P intake restricted the release of FGF23 from bone, there was no increase in renal synthesis of calcitriol, unlike in monogastric species. The reduced FGF23 levels resulted in lower receptor activation, compounded by lower expression of FGFR1(c) receptor in the kidney. This lower expression of FGFR1(c) may account for the lower blood concentration of 24,25(OH)_2_D_3_, consequently reducing the degradation of calcidiol in P-reduced young goats.

## Data availability statement

The original contributions presented in the study are included in the article/supplementary material, further inquiries can be directed to the corresponding author.

## Ethics statement

The animal study was approved by Lower Saxony State Office for Consumer Protection and Food Safety (LAVES). The study was conducted in accordance with the local legislation and institutional requirements.

## Author contributions

LZ: Validation, Visualization, Writing – original draft, Data curation, Formal analysis, Investigation. KH: Formal analysis, Investigation, Visualization, Writing – review & editing. NS: Formal analysis, Investigation, Visualization, Writing – review & editing. FH: Formal analysis, Investigation, Writing – review & editing. MS: Formal analysis, Investigation, Writing – review & editing. GS: Formal analysis, Investigation, Writing – review & editing. AM-B: Writing – review & editing, Conceptualization, Funding acquisition, Project administration, Supervision, Validation, Visualization, Writing – original draft.

## Footnotes

^1^http://blast.ncbi.nlm.nih.gov/Blast.cgi

^2^https://www.moma.dk/normfinder-software

## References

[1] Harmeyer J Martens H. Aspects of urea metabolism in ruminants with reference to the goat. J Dairy Sci. (1980) 63:1707–28. doi: 10.3168/jds.S0022-0302(80)83132-8, PMID: 7451710

[2] Horst RL. Regulation of calcium and phosphorus homeostasis in the dairy cow. J Dairy Sci. (1986) 69:604–16. doi: 10.3168/jds.S0022-0302(86)80445-3, PMID: 3517093

[3] Puggaard L Kristensen NB Sehested J. Effect of decreasing dietary phosphorus supply on net recycling of inorganic phosphate in lactating dairy cows. J Dairy Sci. (2011) 94:1420–9. doi: 10.3168/jds.2010-3582, PMID: 21338807

[4] Muscher A Huber K. Effects of a reduced nitrogen diet on calcitriol levels and calcium metabolism in growing goats. J Steroid Biochem Mol Biol. (2010) 121:304–7. doi: 10.1016/j.jsbmb.2010.03.084, PMID: 20399271

[5] Firmenich CS Elfers K Wilkens MR Breves G Muscher-Banse AS. Modulation of renal calcium and phosphate transporting proteins by dietary nitrogen and/or calcium in young goats. J Anim Sci. (2018) 96:3208–20. doi: 10.1093/jas/sky185, PMID: 29741700 PMC6095294

[6] Behrens JL Schnepel N Hansen K Hustedt K Burmester M Klinger S. Modulation of intestinal phosphate transport in young goats fed a low phosphorus diet. Int J Mol Sci. (2021) 22:866. doi: 10.3390/ijms22020866, PMID: 33467106 PMC7831023

[7] Schröder B Breves G Pfeffer E. Binding properties of duodenal 1,25-dihydroxyvitamin D3 receptors as affected by phosphorus depletion in lactating goats. Comp Biochem Physiol A Comp Physiol. (1990) 96:495–8. doi: 10.1016/0300-9629(90)90668-I, PMID: 1978819

[8] Bergwitz C Jüppner H. Regulation of phosphate homeostasis by PTH, vitamin D, and FGF23. Annu Rev Med. (2010) 61:91–104. doi: 10.1146/annurev.med.051308.111339, PMID: 20059333 PMC4777331

[9] DeLuca HF. Vitamin D: the vitamin and the hormone. Fed Proc. (1974) 33:2211–9. PMID: 4372106

[10] Haussler M Hughes M Baylink D Littledike ET Cork D Pitt M. Influence of phosphate depletion on the biosynthesis and circulating level of 1alpha, 25-dihydroxyvitamin D. Adv Exp Med Biol. (1977) 81:233–50. doi: 10.1007/978-1-4613-4217-5_24, PMID: 899927

[11] Kritmetapak K Kumar R. Phosphate as a signaling molecule. Calcif Tissue Int. (2021) 108:16–31. doi: 10.1007/s00223-019-00636-8, PMID: 31768576 PMC7246150

[12] Sirajudeen S Shah I Al MA. A narrative role of vitamin D and its receptor: with current evidence on the gastric tissues. Int J Mol Sci. (2019) 20:3832. doi: 10.3390/ijms20153832, PMID: 31387330 PMC6695859

[13] Elfers K Liesegang A Wilkens MR Breves G Muscher-Banse AS. Dietary nitrogen and calcium modulate bone metabolism in young goats. J Steroid Biochem Mol Biol. (2016) 164:188–93. doi: 10.1016/j.jsbmb.2015.11.007 26589092

[14] Yoshikawa R Yamamoto H Nakahashi O Kagawa T Tajiri M Nakao M. The age-related changes of dietary phosphate responsiveness in plasma 1,25-dihydroxyvitamin D levels and renal Cyp27b1 and Cyp24a1 gene expression is associated with renal α-klotho gene expression in mice. J Clin Biochem Nutr. (2018) 62:68–74. doi: 10.3164/jcbn.17-20, PMID: 29371756 PMC5773827

[15] Burnett SM Gunawardene SC Bringhurst FR Juppner H Lee H Finkelstein JS. Regulation of C-terminal and intact FGF-23 by dietary phosphate in men and women. J Bone Miner Res. (2006) 21:1187–96. doi: 10.1359/jbmr.060507 16869716

[16] Köhler OM Grünberg W Schnepel N Muscher-Banse AS Rajaeerad A Hummel J. Dietary phosphorus restriction affects bone metabolism, vitamin D metabolism and rumen fermentation traits in sheep. J Anim Physiol Anim Nutr. (2021) 105:35–50. doi: 10.1111/jpn.13449, PMID: 33001513

[17] Hu MC Shiizaki K Kuro-o M Moe OW. Fibroblast growth factor 23 and klotho: physiology and pathophysiology of an endocrine network of mineral metabolism. Annu Rev Physiol. (2013) 75:503–33. doi: 10.1146/annurev-physiol-030212-183727, PMID: 23398153 PMC3770142

[18] Herm G Muscher-Banse AS Breves G Schröder B Wilkens MR. Renal mechanisms of calcium homeostasis in sheep and goats1. J Anim Sci. (2015) 93:1608–21. doi: 10.2527/jas.2014-8450 26020183

[19] Bai X-Y Miao D Goltzman D Karaplis AC. The autosomal dominant hypophosphatemic rickets R176Q mutation in fibroblast growth factor 23 resists proteolytic cleavage and enhances in vivo biological potency. J Biol Chem. (2003) 278:9843–9. doi: 10.1074/jbc.M210490200 12519781

[20] Urakawa I Yamazaki Y Shimada T Iijima K Hasegawa H Okawa K. Klotho converts canonical FGF receptor into a specific receptor for FGF23. Nature. (2006) 444:770–4. doi: 10.1038/nature05315, PMID: 17086194

[21] Andrukhova O Zeitz U Goetz R Mohammadi M Lanske B Erben RG. FGF23 acts directly on renal proximal tubules to induce phosphaturia through activation of the ERK1/2–SGK1 signaling pathway. Bone. (2012) 51:621–8. doi: 10.1016/j.bone.2012.05.015, PMID: 22647968 PMC3419258

[22] Elfers K Wilkens MR Breves G Muscher-Banse AS. Modulation of intestinal calcium and phosphate transport in young goats fed a nitrogen- and/or calcium-reduced diet. Br J Nutr. (2015) 114:1949–64. doi: 10.1017/S000711451500375X, PMID: 26443238

[23] Weber SL Hustedt K Schnepel N Visscher C Muscher-Banse AS. Modulation of GCN2/eIF2&alpha/ATF4 pathway in the liver and induction of FGF21 in young goats fed a protein- and/or phosphorus-reduced diet. Int J Mol Sci. (2023) 24:7153. doi: 10.3390/ijms24087153, PMID: 37108315 PMC10138370

[24] Muscher-Banse A Schröder B Breves G Huber K. Dietary nitrogen reduction enhances urea transport across goat rumen epithelium. J Anim Sci. (2010) 88:3390–8. doi: 10.2527/jas.2010-2949 20581287

[25] Breves G Schröder B. Comparative aspects of gastrointestinal phosphorus metabolism. Nutr Res Rev. (1991) 4:125–40. doi: 10.1079/NRR19910011, PMID: 19094328

[26] National Research Council. Nutrient requirements of small ruminants: sheep, goats, Cervids, and New World camelids. Washington, DC: The National Academies Press (2007).

[27] Ray Sarkar BC Chauhan UPS. A new method for determining micro quantities of calcium in biological materials. Anal Biochem. (1967) 20:155–66. doi: 10.1016/0003-2697(67)90273-4, PMID: 6071917

[28] Kruse-Jarres JD. Klinische Chemie. 2. Spezielle klinisch-chemische Analytik mit Tabellen. New York: G. Fischer Verlag (1979).

[29] Aronov PA Hall LM Dettmer K Stephensen CB Hammock BD. Metabolic profiling of major vitamin D metabolites using Diels-Alder derivatization and ultra-performance liquid chromatography-tandem mass spectrometry. Anal Bioanal Chem. (2008) 391:1917–30. doi: 10.1007/s00216-008-2095-8, PMID: 18437365 PMC3587164

[30] Piechotta M Kedves K Araujo MG Hoeflich A Metzger F Heppelmann M. Hepatic mRNA expression of acid labile subunit and deiodinase 1 differs between cows selected for high versus low concentrations of insulin-like growth factor 1 in late pregnancy. J Dairy Sci. (2013) 96:3737–49. doi: 10.3168/jds.2012-6341, PMID: 23608493

[31] Wilkens MR Elfers K Schmicke M Breves G Muscher-Banse AS. Dietary nitrogen and calcium modulate CYP27B1 expression in young goats. Domest Anim Endocrinol. (2018) 64:70–6. doi: 10.1016/j.domaniend.2018.03.005, PMID: 29754009

[32] Schulze F Malhan D El Khassawna T Heiss C Seckinger A Hose D. A tissue-based approach to selection of reference genes for quantitative real-time PCR in a sheep osteoporosis model. BMC Genomics. (2017) 18:975. doi: 10.1186/s12864-017-4356-4 29258442 PMC5735898

[33] Firmenich CS Schnepel N Hansen K Schmicke M Muscher-Banse AS. Modulation of growth hormone receptor-insulin-like growth factor 1 axis by dietary protein in young ruminants. Br J Nutr. (2020) 123:652–63. doi: 10.1017/S0007114519003040, PMID: 31775916 PMC7025161

[34] Sacco RE Nonnecke BJ Palmer MV Waters WR Lippolis JD Reinhardt TA. Differential expression of cytokines in response to respiratory syncytial virus infection of calves with high or low circulating 25-hydroxyvitamin D3. PLoS One. (2012) 7:e33074. doi: 10.1371/journal.pone.0033074, PMID: 22412984 PMC3297628

[35] Wilkens MR Kunert-Keil C Brinkmeier H Schröder B. Expression of calcium channel TRPV6 in ovine epithelial tissue. Vet J. (2009) 182:294–300. doi: 10.1016/j.tvjl.2008.06.020, PMID: 18701326

[36] Wilkens MR Mrochen N Breves G Schröder B. Gastrointestinal calcium absorption in sheep is mostly insensitive to an alimentary induced challenge of calcium homeostasis. Comp Biochem Physiol B Biochem Mol Biol. (2011) 158:199–207. doi: 10.1016/j.cbpb.2010.11.008 21122820

[37] Starke S Huber K. Adaptive responses of calcium and phosphate homeostasis in goats to low nitrogen intake: renal aspects. J Anim Physiol Anim Nutr. (2014) 98:853–9. doi: 10.1111/jpn.12144, PMID: 24283774

[38] Motulsky HJ Brown RE. Detecting outliers when fitting data with nonlinear regression - a new method based on robust nonlinear regression and the false discovery rate. BMC Bioinformatics. (2006) 7:123. doi: 10.1186/1471-2105-7-123, PMID: 16526949 PMC1472692

[39] Yoshida T Yoshida N Monkawa T Hayashi M Saruta T. Dietary phosphorus deprivation induces 25-hydroxyvitamin D(3) 1alpha-hydroxylase gene expression. Endocrinology. (2001) 142:1720–6. doi: 10.1210/endo.142.5.8119, PMID: 11316734

[40] Breves G Ross R Höller H. Dietary phosphorus depletion in sheep: effects on plasma inorganic phosphorus, calcium, l,25-(OH)_2_-Vit.D_3_ and alkaline phosphatase and on gastrointestinal P and Ga balances. J Agri Sci. (1985) 105:623–9. doi: 10.1017/S0021859600059530

[41] Bikle DD. Vitamin D metabolism, mechanism of action, and clinical applications. Chem Biol. (2014) 21:319–29. doi: 10.1016/j.chembiol.2013.12.016, PMID: 24529992 PMC3968073

[42] Topf JM Murray PT. Hypomagnesemia and hypermagnesemia. Rev Endocr Metab Disord. (2003) 4:195–206. doi: 10.1023/A:1022950321817 12766548

[43] Mouw DR Latessa RA Hickner J. Clinical inquiries. What are the causes of hypomagnesemia? J Fam Pract. (2005) 54:174–6. PMID: 15689296

[44] Bär L Feger M Fajol A Klotz LO Zeng S Lang F. Insulin suppresses the production of fibroblast growth factor 23 (FGF23). Proc Natl Acad Sci USA. (2018) 115:5804–9. doi: 10.1073/pnas.1800160115, PMID: 29760049 PMC5984514

[45] Perwad F Azam N Zhang MY Yamashita T Tenenhouse HS Portale AA. Dietary and serum phosphorus regulate fibroblast growth factor 23 expression and 1,25-dihydroxyvitamin D metabolism in mice. Endocrinology. (2005) 146:5358–64. doi: 10.1210/en.2005-0777, PMID: 16123154

[46] Gattineni J Bates C Twombley K Dwarakanath V Robinson ML Goetz R. FGF23 decreases renal NaPi-2a and NaPi-2c expression and induces hypophosphatemia in vivo predominantly via FGF receptor 1. Am J Physiol Renal Physiol. (2009) 297:F282–91. doi: 10.1152/ajprenal.90742.2008, PMID: 19515808 PMC2724258

[47] Muñoz-Castañeda JR Herencia C Pendón-Ruiz de Mier MV Rodriguez-Ortiz ME Diaz-Tocados JM Vergara N. Differential regulation of renal klotho and FGFR1 in normal and uremic rats. FASEB J. (2017) 31:3858–67. doi: 10.1096/fj.201700006R, PMID: 28515153

[48] Andrukhova O Smorodchenko A Egerbacher M Streicher C Zeitz U Goetz R. FGF23 promotes renal calcium reabsorption through the TRPV5 channel. EMBO J. (2014) 33:229–46. doi: 10.1002/embj.201284188 24434184 PMC3983685

[49] Han X Yang J Li L Huang J King G Quarles LD. Conditional deletion of Fgfr1 in the proximal and distal tubule identifies distinct roles in phosphate and calcium transport. PLoS One. (2016) 11:e0147845. doi: 10.1371/journal.pone.0147845, PMID: 26839958 PMC4739706

[50] Forster RE Jurutka PW Hsieh J-C Haussler CA Lowmiller CL Kaneko I. Vitamin D receptor controls expression of the anti-aging klotho gene in mouse and human renal cells. Biochem Biophys Res Commun. (2011) 414:557–62. doi: 10.1016/j.bbrc.2011.09.117, PMID: 21982773 PMC3209523

[51] Ohyama Y Ozono K Uchida M Yoshimura M Shinki T Suda T. Functional assessment of two vitamin D-responsive elements in the rat 25-hydroxyvitamin D3 24-hydroxylase gene. J Biol Chem. (1996) 271:30381–5. doi: 10.1074/jbc.271.48.30381 8940000

[52] Mazzaferro S Pasquali M Pirro G Rotondi S Tartaglione L. The bone and the kidney. Arch Biochem Biophys. (2010) 503:95–102. doi: 10.1016/j.abb.2010.06.028 20599669

